# Tamsulosin Versus Mirabegron in Relieving Ureteric Stent-Related Symptoms: A Prospective, Double-Blinded, Randomized Controlled Trial

**DOI:** 10.7759/cureus.50502

**Published:** 2023-12-14

**Authors:** Mohamed Javid, Atif Abdullah, Ramesh Ganapathy, Yogendra Basoo Gupta, Sudhakaran Selvaraj, Ananda Kumar Ilangovan, Senthilkumar Sivalingam, Srikala Prasad

**Affiliations:** 1 Urology, Chengalpattu Medical College, Chengalpattu, IND; 2 Urology, AR Hospital, Ramanathapuram, IND; 3 Urology, FH Medical College, Agra, IND

**Keywords:** ureteral stent symptoms questionnaire, stent-related symptoms, ureteral stent, mirabegron, tamsulosin

## Abstract

Introduction

Alpha-adrenergic blockers like tamsulosin are widely used in the treatment of stent-related symptoms due to ureteric stents. Recently, mirabegron has emerged as a potential alternative. So, our study aimed to compare the effect of mirabegron and tamsulosin on ureteric stent-related morbidity.

Methods

In this randomized controlled study, 80 patients undergoing uncomplicated ureteroscopic lithotripsy with double J stenting for ureteric stones were enrolled. They were divided into two groups: Group A (n=40) received mirabegron (25mg) and Group B (n=40) received tamsulosin (0.4mg). Outcomes were assessed using the Ureteral Stent Symptom Questionnaire (USSQ), International Prostate Symptoms Score (IPSS), and the visual analog pain scale. The t-test and the Chi-square test were utilized to study the efficacy of the interventions across both groups.

Results

The USSQ urinary symptom score (25.5 vs 33.45; p < 0.001) and body pain score (16.15 vs 26.02; P < 0.001) were significantly lower in the mirabegron group. However, the general health score (17.0 vs 17.28; p = 0.62) and work performance score (7.6 vs 8.0; p = 0.28) did not show a significant difference. The storage symptom score was significantly lower in the mirabegron group (3.98 vs 5.1; p = 0.001). Furthermore, the mirabegron group reported a better quality of life score (2.18 vs 3; p < 0.001).

Conclusion

Mirabegron has been shown to reduce urinary symptoms associated with ureteric stents and also results in a better quality of life when compared with tamsulosin. However, large-scale, prospective, multicentric studies are further required to holistically evaluate and comprehend the beneficial effects of mirabegron on stent-related morbidity.

## Introduction

Ureteral stents have long been an integral component in the armamentarium of a urologist [1,2]. A few common indications for ureteral stent placement include the relief of obstructions, aiding the recovery of ureteric injuries, and routine adjuncts following surgeries [1-4]. Their applications have been diversified and are constantly widening since their advent four decades ago when Zimskind et al. [5,6] first described a cystoscopically placed temporary ureteral stent.

However, despite the perceived benefits, the placement of ureteral stents often causes a constellation of symptoms that have been collectively termed stent-related symptoms (SRS) [7]. Their incidence ranges from 19 to 76% and includes a broad spectrum of symptoms such as lower urinary tract symptoms (such as frequency, urgency, incomplete emptying), dysuria, flank pain, suprapubic pain, urinary incontinence, and even hematuria [7]. To methodically measure the SRS, Joshi et al. developed a validated tool known as the Ureteral Stent Symptoms Questionnaire (USSQ) [4]. This tool is a safe psychometric instrument that comprehensively gauges the impact of ureteral stents, with a focus on both patients' symptoms and the quality-of-life implications. In addition, the International Prostate Symptom Score (IPSS), a validated tool conventionally used in the evaluation of lower urinary tract symptoms (LUTS), has also been employed to study SRS [7-10].

To alleviate SRS, various strategies have been explored, including pharmacological interventions, alterations in stent design, and the utilization of drug-eluting stents [7]. Among these, the usage of drugs to address this morbidity has been widely practiced. Tamsulosin, a selective alpha 1a blocker, has long been used and has robust evidence supporting its efficacy in mitigating SRS [3,5,7,10-13]. Recently, mirabegron, a beta-3 agonist with wide usage in overactive bladder (OAB), has been investigated for its application in the management of SRS [2,3]. However, there is a paucity of direct comparative studies between mirabegron and tamsulosin in this regard. So, our study aimed to compare and analyze the efficacy of tamsulosin and mirabegron in the management of SRS using both the USSQ and IPSS.

## Materials and methods

Study design

A prospective, double-blinded, randomized controlled trial was conducted over nine months. The study was approved by the Institutional Ethical Committee of Chengalpattu Medical College (No: CMCH - 19 - PR - 092). Also, the study was registered with the Clinical Trials Registry - India (Reg No: CTRI/2021/01/030340). The block randomization method was utilized to divide the participants into two groups.

Study participants

**Figure 1:**
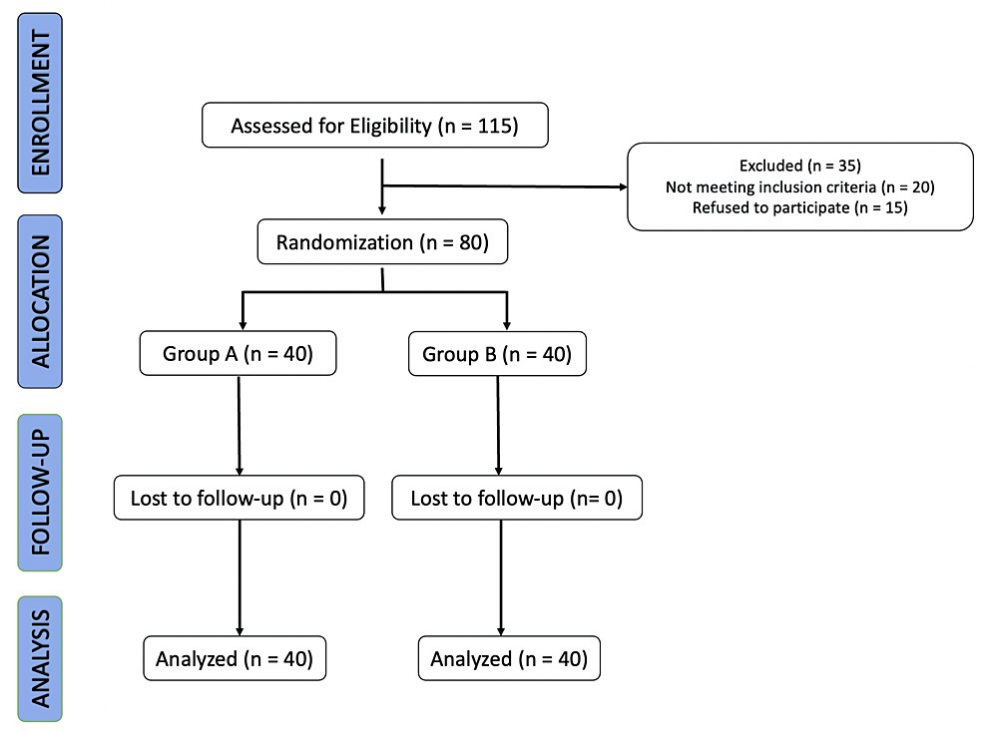
CONSORT diagram of the study design Group A: Tamsulosin group; Group B: Mirabegron group

A total of 80 patients were enrolled in our study after obtaining proper informed consent from each participant. We included all patients who underwent unilateral ureteroscopic lithotripsy (URSL) with ureteral stent (4 Fr, 26 cm polyurethane Double J stent) placement for uncomplicated ureteric calculi. The patients who were excluded from the study were those who had incomplete clearance of stone, major complications during or after ureteroscopy (avulsion or perforation), bilateral stent insertion, concomitant urinary tract infection, children (<18 years of age), overactive bladder syndrome, neurogenic bladder, any history or current treatment for urge/stress mixed incontinence and chronic pelvic pain syndrome. A semi-rigid ureteroscope with a pneumatic lithotripter was used for lithotripsy. An X-ray was taken on the second postoperative day to ensure and confirm the stent’s correct position. To standardize the study all patients had their stents removed by cystoscopy on the 15th postoperative day. The patients were divided into two groups: Group A (Tamsulosin group) and Group B (Mirabegron group) (Figure 1). All patients in their respective groups received uniform treatment after URSL (Table 1).

**Table 1:** Treatment received by the patients after URSL from the first postoperative day Group A: Tamsulosin group; Group B: Mirabegron group; URSL: Ureteroscopic lithotripsy

Group A	Group B
Tablet Ciprofloxacin 500 mg twice daily for three days.	Tablet Ciprofloxacin 500 mg twice daily for three days.
Tablet Paracetamol 500 mg thrice daily for three days.	Tablet Paracetamol 500 mg thrice daily for three days.
Tablet Tamsulosin 0.4 µg once daily for two weeks.	Tablet Mirabegron 25 mg once daily for two weeks.

Data collection

Patients were thoroughly assessed using the following to assess the effectiveness.

Ureteral Stent Symptom Questionnaire (USSQ)

This is a validated questionnaire that evaluates the symptoms by categorizing them into the following domains: urinary symptoms, body pain, general health, work performance, and sexual matters [4].

International Prostate Symptom Score (IPSS)

This is a validated questionnaire that evaluates the symptoms by categorizing them into three groups: storage symptoms, voiding symptoms, and quality of life [7-10].

Visual Analog Pain Scale

All assessments were conducted at the time of stent removal, which was scheduled on the fifteenth postoperative day.

Statistical analysis

The data were entered into Microsoft Excel and analyzed using SPSS Statistics for Windows, Version 25 (Released 2017; IBM Corp., Armonk, New York, United States). Descriptive statistics (including means, standard deviations, frequencies, and percentages) were utilized to summarize the categorical and quantitative variables. The efficacy of the interventions across both groups was evaluated using the t-test and the Chi-square test. The p-values of ≤ 0.05 were considered statistically significant and 95% confidence intervals were reported to indicate the precision of the results.

## Results

A total of 80 patients (Group A: Males - 27, Females - 13; Group B: Males - 26, Females - 14) were enrolled and patients completed the study with no one lost to follow-up. The mean age of the participants in years in Group A was 44.07 (±12.09) and in Group B was 40.05 (±11.77). Table 2 details the location and side of the ureteric calculus in both groups.

**Table 2:** Location and side of the ureteric calculus Group A: Tamsulosin group; Group B: Mirabegron group

Site of the calculus	Group A	Group B	Total
Left Upper ureteric calculus	9	10	19
Left Lower ureteric calculus	6	9	15
Right Lower ureteric calculus	13	10	23
Right Upper ureteric calculus	12	11	23
Total	40	40	80

Comparing Group A with Group B using the USSQ, only the urinary symptoms (p < 0.001) and body pain (p < 0.001) showed significant differences (Table 3). 

**Table 3:** USSQ scores Group A: Tamsulosin group; Group B: Mirabegron group; USSQ: Ureteral Stent Symptom Questionnaire

USSQ	Group	N	Mean	SD	t	p
Urinary symptom	A	40	33.45	6.089	6.807	0.0001
	B	40	25.5	4.182		
Body Pain	A	40	26.02	4.282	11.058	0.0001
	B	40	16.15	3.683		
General Health	A	40	17.28	2.582	0.51	0.62
	B	40	17.0	2.276		
Work Performance	A	40	8.0	0.96	1.09	0.28
	B	40	7.62	1.97		
Sexual Matters	A	40	3.75	1.581	1.4	0.165
	B	40	3.28	1.45		

When utilizing the IPSS and VAS for comparison between the two groups, statistically significant differences were observed in the storage symptom domain (p < 0.001) and quality of life aspect (p < 0.001) of the IPSS. However, there was no significant difference in the visual analogue pain score (Table 4).

**Table 4:** IPSS and visual analogue pain score Group A: Tamsulosin group; Group B: Mirabegron group; IPSS: International Prostate Symptom Score

IPSS / Visual Analogue Pain	Group	Mean	SD	t	p
Storage symptom score	A	5.1	1.582	3.443	0.001
	B	3.98	1.33		
Voiding symptom score	A	1.92	1.047	1.034	0.304
	B	2.15	0.893		
Visual analogue pain score	A	2.5	0.961	0.473	0.637
	B	2.4	0.928		
Quality of life	A	3	0.934	4.215	0.0001
	B	2.18	0.813		

## Discussion

The significance of ureteral stents in endourological interventions remains undisputed and their utilization will probably continue to rise [2]. They serve a multitude of purposes such as preventing the obstruction of urinary flow in the ureter due to mucosal edema and can also facilitate the healing of the mucosa following a complicated procedure [1,2]. Ureteral stents can passively dilate the ureter, which can subsequently aid in the passage of residual stones [1,2].

Despite the myriad benefits that ureteral stents offer, stent-related morbidity poses a significant concern. A concerning array of SRS plagues a substantial proportion of patients, casting shadows on the overall patient experience. Fifty-eight percent of patients reported reduced work performance, 32% reported sexual dysfunction, and 80% reported LUTS and pain that is attributable to ureteral stents [2]. With the sustained and increasing use of ureteral stents, addressing these symptoms is of paramount importance [2].

Various strategies have been employed to alleviate the SRS such as the development of newer stents, coatings, and biomaterials that have been specifically developed for this purpose [2,3]. But still, the development of the ideal stent remains elusive [2,3]. Currently, the most effective modality to manage SRS is pharmacological therapy which includes the use of analgesics, anticholinergics, and alpha blockers [3,5,7,10-15]. Among these alpha-blockers, in particular, tamsulosin has been extensively studied and shown to be effective in improving SRS [3,5,7,10-15].

Though the precise mechanism causing the SRS remains an enigma, various potential pathogenesis have been reported [3]. They include trigonal irritation, urinary reflux secondary to the stent, and ureteric spasms [3]. Alpha-blockers decrease the bladder outlet resistance, and this can lead to the decrease of voiding pressure and could probably decrease reflux [3,5,16]. Also, the alpha-1 adrenergic receptors are present throughout the ureter with increased concentration at the distal aspect and hence blocking these alpha receptors could decrease the ureteral spasms [3,5,16]. These mechanisms of alpha-blockers could reduce SRS.

The smooth muscles and urothelium of the ureters express beta-adrenergic receptors and they cause relaxation of the ureters [2,3]. So, it has been reported that the usage of beta-adrenergic agonists can relax the ureter and mitigate the ureteric spasm [2,3]. Furthermore, beta-3 agonists cause bladder relaxation and decrease bladder contraction, and this could prevent reflux and lead to a decrease in ureteral and renal pelvic pressures [2,3]. Also, since the SRS parallels those of OAB, mirabegron was suggested to reduce SRS and has been explored [3].

Tae et al. first investigated mirabegron as a beta-3 agonist in the management of SRS and found that mirabegron decreased pain (21.96 vs 13.96; p = 0.007) and overall pain (5.58 vs 2.83; p = 0.002) but did not improve USSQ urinary symptom scores (32.58 vs 27.92, p = 0.582) or USSQ general health scores [3]. They concluded that the usage of once-daily mirabegron could potentially lead to a reduction in ureteric stent-related discomfort [3]. The study by Yavuz et al. compared mirabegron against tamsulosin along with a control group and they found that the need for analgesics was lower in the mirabegron group in comparison with the control group, but the USSQ score including the urinary symptoms score (27.8 vs 24.5, p = 0.423) was not different between the mirabegron and control groups [2]. They concluded that though mirabegron can reduce the analgesic need they don't significantly improve SRS [2]. In our study, we found out that mirabegron was better than tamsulosin in relieving urinary symptoms (33.45 vs 25.5; p < 0.001) and body pain (26.02 vs 16.15; p < 0.001). Furthermore, in comparison with tamsulosin, mirabegron also improved the storage symptoms (p < 0.001) and quality of life aspect (p < 0.001) of the IPSS.

The findings of our trial must be considered within the context of some limitations. Firstly, our study was conducted with a limited sample size and was done in a single center. So, future research should consider a well-structured multicentric approach with larger participants to offer more comprehensive insights. Secondly, the follow-up duration in our study was confined to 15 days. For a more holistic comprehension of the long-term effects of mirabegron and tamsulosin on SRS, extending the follow-up period in subsequent studies is recommended.

## Conclusions

In our study, we found that mirabegron appears to be more effective than tamsulosin in reducing urinary symptoms and body pain in patients with ureteral stents. Patients treated with mirabegron also reported a better overall quality of life than those on tamsulosin. Hence, mirabegron can be used in the management of ureteral stent-related symptoms. However, large multicentric studies are needed to fully elucidate and understand mirabegron's benefits in ureteral stent-related morbidity.
